# Response to noninvasive home mechanical vEntilation in Myotonic Dystrophy type 1: The multicenter REMeDY study

**DOI:** 10.1177/22143602261428053

**Published:** 2026-02-26

**Authors:** Bettine AH Vosse, Leandre André la Fontaine, Nicolle Cobben, Danielle Kock-Cordeiro, Anda Hazenberg, Michael Gaytant, PJ Wijkstra, Catharina Faber

**Affiliations:** 1 Department of Pulmonary Diseases and Home Mechanical Ventilation, 199236 Maastricht University Medical Center+, Maastricht, the Netherlands; 2 School for Mental Health and Neuroscience (MHeNS), Maastricht University, Maastricht, the Netherlands; 3 Department of Neurology, Maastricht University Medical Center+, Maastricht, the Netherlands; 4 Department of Intensive Care and Home Mechanical Ventilation, 6993 Erasmus University Medical Center, Rotterdam, the Netherlands; 5 Department of Pulmonary Diseases and Home Mechanical Ventilation, 10173 University of Groningen, University Medical Center Groningen, Groningen, the Netherlands; 6 Department of Pulmonary Diseases and Home Mechanical Ventilation, 8124 University Medical Center Utrecht, Utrecht, the Netherlands; 7 University of Groningen, University Medical Center Groningen, Groningen Research Institute for Asthma and COPD, Groningen, the Netherlands

**Keywords:** myotonic dystrophy, noninvasive ventilation, respiratory insufficiency, respiration, ventilators, patient adherence

## Abstract

Myotonic dystrophy type 1 (DM1) frequently leads to chronic respiratory failure, yet the effectiveness of noninvasive home mechanical ventilation (HMV) remains understudied. Our objective was to assess the effects of HMV on gas exchange, health-related quality of life (HRQL), and daily functioning, and to explore baseline predictors of treatment response. A prospective multicenter study was conducted in which clinical data, pulmonary function tests, blood gas analysis, polysomnography and overnight pulse oximetry with transcutaneous CO_2_ monitoring, and validated questionnaires were collected at baseline and after six months of treatment. Paired t-tests and correlation analyses assessed treatment outcomes and predictive factors. Forty participants (mean age 46.6 years, 63% male) were enrolled, and follow-up data of 38 participants were available for analysis. After six months of treatment, significant improvements in gas exchange were observed with a reduction in daytime pCO_2_ of 0.48 ± 0.81 kPa (p = 0.001) and nocturnal mean pCO_2_ of 0.92 ± 0.81 kPa (p < 0.001). HRQL, assessed with the Severe Respiratory Insufficiency (SRI) questionnaire, improved significantly, with a mean increase of 9.9 ± 8.9 points (p < 0.001).No significant changes were seen in daily functioning measured with the DM1-Activ^C^ questionnaire. Improvements in nocturnal pCO_2_ correlated with HRQL gains (r = 0.427, p < 0.021), while baseline characteristics were not predictive of response. This prospective multicenter study demonstrates that nocturnal HMV is associated with significant improvements in gas exchange and HRQL in patients with DM1 over six months.

## Introduction

Myotonic dystrophy type 1 (DM1) is the most common form of adult-onset muscular dystrophy with a population-based estimated prevalence of 1 in 2.100, and with a reduced life expectancy of 54–60 years for adult-onset DM1.^1–3^ Respiratory failure is the most frequent cause of death and is due to a complex mechanism of decreased respiratory muscle strength, reduced central respiratory drive, diminished chest wall compliance, and upper airway obstruction.^2,4,5^ Home mechanical ventilation (HMV), encompassing both invasive and noninvasive ventilation, is presumed to be an effective treatment for chronic respiratory failure in DM1. In clinical practice, HMV in DM1 is most often delivered as noninvasive ventilation. However, studies evaluating the effectiveness of noninvasive HMV in this population are scarce and mainly observational designed without predefined outcome measurements.^6^ Randomized controlled trials or prospective studies are lacking. In addition, it is known that HMV is not successful in every DM1 patient, and varies greatly from a very burdensome experience in some, to a highly beneficial treatment in others. Patient selection and treatment adherence remain challenging despite consensus-based recommendations which are completely based on expert opinion rather than backed up by empirical evidence.^7^ There is no general agreement on the precise definition of treatment success with regard to HMV. Prolonged survival, reduction of carbon dioxide levels (pCO_2_) and improvement of oxygenation are regarded as important aims to achieve when treating patients with HMV.^8^ On the other hand, from a patient's perspective it might be more important to focus on symptom relief, and enhancing or maintaining quality of life and daily functioning. While the pathophysiological pathway from neuromuscular weakness to chronic respiratory failure is largely shared across neuromuscular diseases, important disease-specific differences exist. Evidence from disorders such as amyotrophic lateral sclerosis and Duchenne muscular dystrophy shows that noninvasive HMV improves gas exchange, symptoms, and survival.^9,10^ However, the multisystem nature of DM1, including cognitive impairment and excessive daytime sleepiness, may influence treatment adherence and perceived benefit, underscoring the need for DM1-specific prospective data.^11^ Accordingly, in this prospective multicenter study, we aimed to evaluate the effect of noninvasive HMV on gas exchange, quality of life, and daily functioning in DM1 patients following six months of treatment with HMV. A secondary objective was to explore associations between baseline clinical characteristics and treatment response to HMV.

## Methods

A prospective multicenter study was conducted at the four centers of HMV in the Netherlands: Maastricht, Rotterdam, Groningen and Utrecht. These four centers are responsible for all Dutch patients who need HMV. The local Medical Ethics Committee of the Maastricht University Medical Center+ (MUMC+) concluded that the study protocol falls outside the scope of the Medical Research Involving Human Subjects Act (registration number METC2018-0853). The study was conducted according to the applicable research principles. Written informed consent was obtained from all included participants. All adult DM1 patients with an indication to start with noninvasive HMV were asked to participate. HMV indication and inclusion into this study was in accordance with the current consensus-based care recommendations for DM1 patients: at least one or more daytime or nighttime symptoms suggestive of chronic respiratory failure in combination with daytime hypercapnia (PaCO_2_ ≥ 6.0 kPa) or evidence of nocturnal hypoventilation (transcutaneous PCO_2_ > 6.7 kPa for >50% of total sleep time).^7^ Exclusion criteria included: previous treatment with Continuous Positive Airway Pressure (CPAP) or HMV in the past five years; other condition leading to hypercapnia; heart failure New York Heart Association (NYHA) classification 4; unstable angina pectoris.

Prior to initiation of HMV, extensive data was collected to characterize the study population. Demographics included CTG (cytosine-thymine-guanine) repeat length, and neurological characterization: the ordinal Medical Research Council (MRC) sum score was used to test muscular strength with a higher score indicating higher muscular strength.^12^ The Muscular Impairment Rating Scale (MIRS), a DM1-specific scale for muscle weakness, was used to rate muscular impairment.^13^ To assess the pulmonary function, spirometry was performed in every patient (upright sitting and supine). All pulmonary function tests met appropriate standards of the European Respiratory Society/American Thoracic Society.^14^ Arterial blood gas analysis was used to assess daytime hypercapnia. When an arterial blood gas was not available, a capillary blood gas was used as an alternative.^15^ In order to detect sleep apnea and/or nocturnal hypoventilation, an attended polysomnography (BrainLab RT and Morpheus, Natus Group (OSG) Kontich, Belgium) was performed in a subset of participants in a sleep laboratory, while overnight pulse oximetry with transcutaneous CO_2_ monitoring (Sentec, Therwil, Switzerland) was performed at the patient's home in the entire cohort. Apnea–hypopnea index values were obtained from attended polysomnography in a subset of patients at baseline. Follow-up AHI values were obtained from attended polysomnography, depending on availability.

A selection of questionnaires was filled out prior to HMV initiation. The DM1-Activ^C^ reflects the patients’ daily activity and social participation with 100 points representing the best possible score and 0 points representing the worst possible score indicating a total limitation in activity and participation.^16^ The Severe Respiratory Insufficiency (SRI) questionnaire was used to measure health-related quality of life (HRQL) with a score between 0 and 100 with higher values indicating a better HRQL.^17,18^ The Fatigue and Daytime Sleepiness Scale (FDSS) measures symptoms of fatigue and daytime sleepiness with a score of 0 indicating no fatigue and daytime sleepiness, and 100 indicating severe fatigue and daytime sleepiness.^19^ A visual analogue scale was used to measure perceived health on a scale of 0–100 with a higher number representing a better perceived health (EQ-VAS health). The Hospital Anxiety and Depression Scale (HADS) was used to assess emotional functioning with a maximum score of 21 and scores of 8 or more are indicative of the possible presence of anxiety or depression.^20,21^ The Care Dependency Scale (CDS) was used to determine the extent to which a patient is dependent on others for everyday tasks and self-care with a total score ranging from 15 (totally dependent) to 75 (totally independent).^22^ The Caregiver Strain Index (CSI) identifies strain among caregivers with a higher score indicating higher strain (maximum score of 13).^23^

Patients were initiated on HMV at home or during a planned hospital stay, under close supervision of specialized physicians and nurses of the HMV center. Both situations are interchangeable as it was shown that initiation at home is not inferior compared to initiation at a hospital.^24^ Patients were followed according to a standardized clinical follow-up protocol, including early telephone contact by a specialized ventilation nurse approximately two weeks after initiation, an outpatient visit after 6–12 weeks, and a comprehensive evaluation at six months. Follow-up included assessment of symptoms, ventilator use and adherence, blood gas analysis, and adjustment of ventilator settings as needed. Additional telephone contacts were provided for patients with suboptimal adherence. At six months, next to spirometry, blood gas analysis, and polysomnography or overnight pulse oximetry with transcutaneous CO_2_ monitoring, data were collected on treatment adherence with the ventilator, and ventilator settings. Treatment adherence was assessed using ventilator software downloads, reflecting device use over the preceding six-month period (approximately 180 days). The previously mentioned questionnaires (DM1-Activ^C^, FDSS, EQ-VAS health, HADS, SRI, CSI, CDS)were filled in with the addition of the S^3^-NIV questionnaire. The S^3^-NIV questionnaire was used to determine HMV-related side effects, symptoms and sleep quality. The lowest possible score (0) corresponds to the highest impact of disease and treatment, while the highest possible score (10) corresponds to the lowest impact of disease and treatment.^25,26^ Also, patients were interviewed using a standardized set of study-specific questions addressing perceived benefit, tolerability, and side effects of noninvasive ventilation. Responses were recorded in a structured format.

### Statistical analysis

Statistical analysis was performed using IBM SPSS statistics software version 28. Continuous variables were expressed as mean with standard deviation or as median with 1^st^ and 3^rd^ quartile in case of skewed distribution. Categorical variables were expressed as count and percentage. Paired samples t-test was performed to compare body mass index (BMI), pulmonary function parameters, gas exchange parameters and parameters on quality of life and daily functioning before and after six months of treatment. Associations between baseline characteristics, treatment adherence, and treatment response were explored using bivariate analyses. These analyses were predefined as exploratory, and no formal adjustment for multiple comparisons was applied. A p-value of <0.05 was considered statistically significant.

## Results

**Figure 1.**
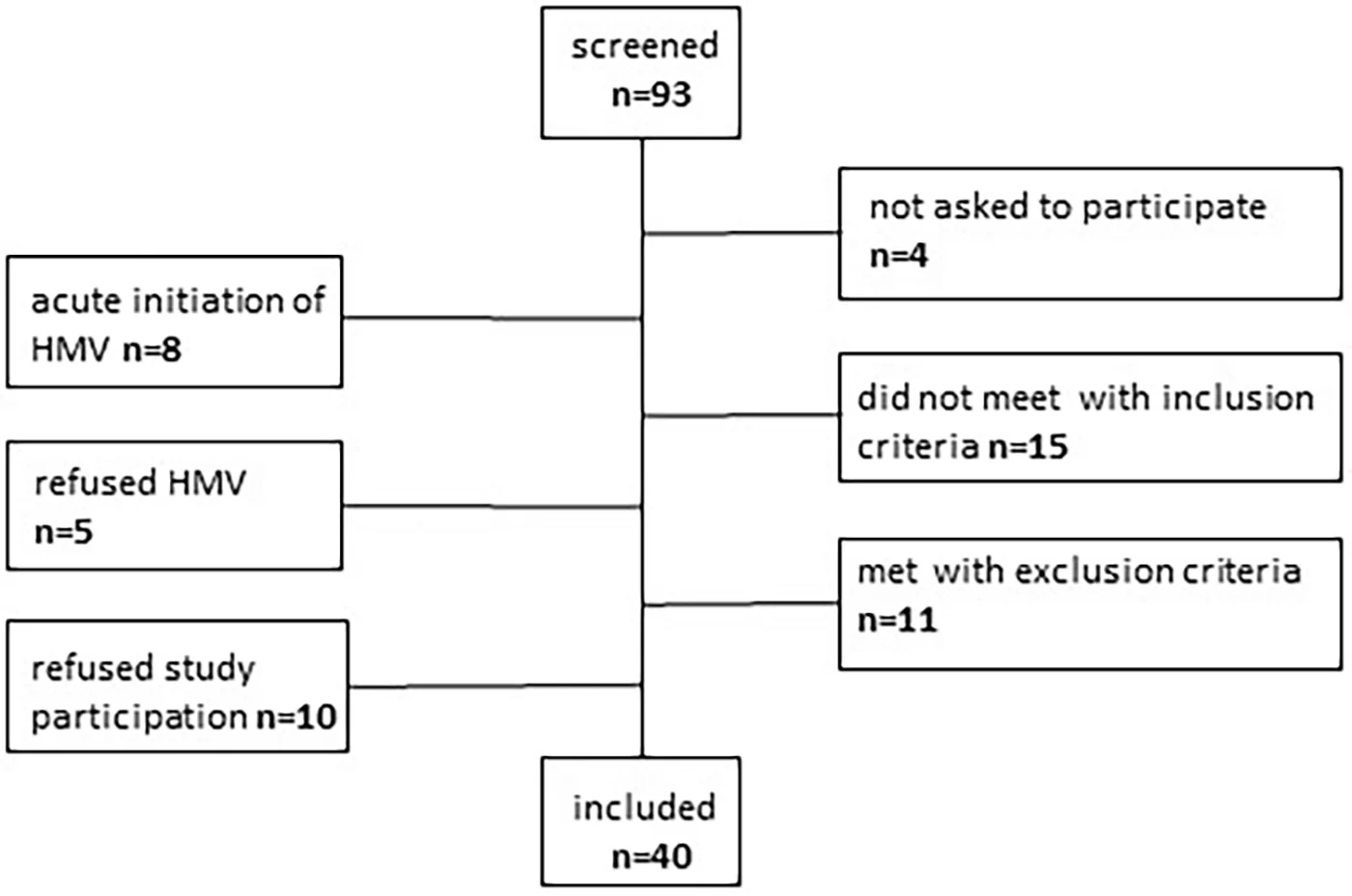
Flowchart of the screening process.

Between August 2019 and September 2023, 93 patients were screened for eligibility, and 40 patients were included in the study. A flow chart of the screening procedure can be found in Figure 1.

Home initiation of HMV was done in 19 patients, while 21 patients started HMV during a scheduled hospital stay. Indication for HMV was daytime hypercapnia (and nocturnal hypoventilation) in 28 patients and nocturnal hypoventilation in 12 patients. Due to difficulty in including enough patients, we decided to abandon the exclusion criterion of previously treated with CPAP or HMV halfway through the inclusion period. Prior to this adjustment, 6 patients were excluded because they used CPAP. After modification of the protocol, five patients were included that had been previously treated with CPAP or were still using CPAP at the time of inclusion. The baseline characteristics of the study sample can be found in Table 1. The CTG repeat length was known in 23 patients, the other 17 patients had been tested positive in the past, but the exact CTG repeat length could not be retrieved. The majority of patients had the adult subtype of DM1 (n = 33), the other patients had the juvenile subtype (n = 5) or congenital subtype (n = 2). Twenty-three patients underwent a PSG with a diagnosis of sleep apnea in 20 patients (87%). One patient died during the follow-up period (cause of death: pancreatitis) and was excluded from the response analysis at 6 months. Another patient had to be excluded from the response analysis because of missing data at 6 months.

**Table 1.** Characteristics at baseline and response parameters at 6 months of treatment.

	Baseline	At 6 months	P
Age	46.6 ± 14.4		
Male	25 (63%)		
BMI (n = 32)	25.9 ± 6.5	25.0 ± 6.4	0.665
MRC sum score on muscle strength	93.0 ± 12.6		
MIRS	4 [3–4]		
FVC sitting (% of predicted) (n = 33)	57.1 ± 21.2	59.2 ± 22.2	0.504
FVC supine (% of predicted) (n = 23)	50.6 ± 24.7	52.7 ± 26.2	0.088
Peak cough flow (L/min) (n = 20)	221.0 ± 69.2	214.1 ± 88.9	0.357
Daytime blood gas pCO_2_ (kPa) (n = 36)	6.5 ± 0.75	6.1 ± 0.69	0.001*
Apnea hypopnea index (events/h) (n = 16)	25.6 ± 19.3	5.2 ± 5.3	<0.001*
Nocturnal mean SpO_2_ (%) (n = 32)	91.8 ± 4.2	93.3 ± 3.6	0.001*
Nocturnal mean pCO_2_ (kPa) (n = 31)	6.6 ± 0.73	5.7 ± 0.88	<0.001*
Maximum nocturnal pCO_2_ (kPa) (n = 33)	7.9 ± 1.1	6.7 ± 1.0	<0.001*

BMI = body mass index; MRC = Medical Research Council; MIRS = muscular impairment rating scale; FVC = forced vital capacity; pCO_2_ = partial pressure of CO_2_; SpO_2_ = peripheral oxygen saturation; data expressed as mean ± standard deviation, frequency (%), or median [IQR]; sample size varied per variable due to missing data; the sample sizes (n) reported in the table refer to patients with available paired baseline and six-month follow-up data. *p < 0.05.

After six months of treatment with nocturnal HMV, significant improvements in gas exchange were observed both during daytime and at night (Table 1). Daytime pCO_2_ decreased by 0.48 ± 0.81 kPa (p = 0.001), while nocturnal mean pCO_2_ decreased by 0.92 ± 0.81 kPa (p < 0.001). Mean nocturnal SpO_2_ increased by 1.78 ± 2.83 percentage points (p < 0.001). In addition, HRQL, assessed with the SRI questionnaire, improved significantly, with a mean increase of 9.9 ± 8.9 points after six months of HMV (p < 0.001; Table 2).

**Table 2.** Questionnaires at baseline and response at 6 months of treatment.

	Baseline	At 6 months	P
DM1-Activ (n = 34)	53.8 ± 17.3	55.0 ± 18.7	0.981
SRI sum score (n = 34)	57.2 ± 7.4	67.1 ± 10.5	<0.001
FDSS (n = 34)	46.5 ± 9.6	44.4 ± 9.4	0.112
EQ-VAS health (n = 31)	61.5 ± 17.6	62.6 ± 19.5	0.401
HADS anxiety (n = 34)	3.4 ± 2.2	3.5 ± 2.6	0.793
HADS depression (n = 38)	4.5 ± 2.7	3.9 ± 3.0	0.393
CDS (n = 34)	62.8 ± 11.6	62.3 ± 11.3	0.328
CSI (n = 14)	7.4 ± 3.1	7.6 ± 3.3	0.625
S^3^-NIV (n = 35)		6.7 ± 1.5	

FDSS = Fatigue and Daytime Sleepiness Scale; SRI = Severe Respiratory Insufficiency questionnaire; EQ-VAS = EuroQol-visual analogue scales; HADS = Hospital Anxiety and Depression Scale; CDS = Care Dependency Scale; S^3^-NIV = S^3^ noninvasive ventilation questionnaire; data expressed as mean ± standard deviation; sample size varied per variable due to missing data; the sample sizes (n) reported in the table refer to patients with available paired baseline and six-month follow-up data.*p < 0.05.

The ventilator settings and data on treatment adherence after six months can be found in Table 3. Data on treatment adherence were lacking in 3 patients. The following ventilators were used: Löwenstein Prisma VENT50-C, Bad Ems, Germany (n = 18), ResMed Stellar^TM^ 150, Didcot, United Kingdom (n = 10), Philips Respironics A40, Eindhoven, the Netherlands (n = 9) and Philips Respironics Trilogy 100, Eindhoven, the Netherlands (n = 3). Almost all patients used a full face mask with the exception of three patients who used a nose mask. Data on treatment adherence at six months were available for 33 patients, while data on average nightly use were available for 35 patients (Table 3). Missing adherence data were primarily due to technical issues, including unavailable or corrupted SD cards and differences in data formats between ventilator devices. Over the six-month follow-up period, 10 patients discontinued the use of noninvasive ventilation.

**Table 3.** Ventilator settings and treatment adherence after 6 months of treatment.

IPAP (cmH_2_O)	14.7 ± 3.0
EPAP (cmH_2_O)	6.1 ± 1.4
Back up rate per minute	12.7 ± 2.4
Days used as percentage of total days^a^	66.3 ± 39.1
0–30%	8
31–79%	7
>80%	18
Average use per night	
0–2h	8
2–4h	5
>4h	22

IPAP = inspiratory positive airway pressure; EPAP = expiratory positive airway pressure.

^a^ Percentages indicate days with ventilator use over ∼180 days.

After six months, 34 patients were interviewed and answered questions on effect, tolerability and side effects of HMV (Table 4). Of the total group, 28 out of 40 patients (70%) were motivated to continue the use of HMV. The reasons for continuation were: ‘because I feel better’ (n = 19), ‘because it is good for my health’ (n = 27), ‘because the doctor tells me to do’ (n = 11). Other reasons were: ‘because I feel less short of breath’, ‘because my headaches disappeared’ (n = 2), ‘because I participate in this research’ (n = 2). The patients that discontinued HMV gave as reasons: ‘no benefit from the treatment’ (n = 5), ‘unable to sleep with HMV’ (n = 9), ‘mask problems’ (n = 3).

**Table 4.** Tolerability and side effects after 6 months of treatment.

	Yes	Moderate	No
Fall asleep with HMV	21 (62%)	7 (21%)	6 (17%)
Sleep all night through with HMV	14 (41%)	11 (32%)	9 (27%)
Tolerate HMV all night	21 (62%)		13 (38%)
Problems with using HMV	3 (9%)		31 (91%)
Alarms	6 (18%)		28 (82%)
Technical problems	2 (6%)		32 (94%)
Mask leakage	6 (18%)	10 (29%)	18 (53%)
Mask decubitus	2 (6%)	14 (41%)	18 (53%)
Dry mouth	15 (50%)		15 (50%)
Nose obstruction	4 (14%)		25 (86%)
Aerophagia	3 (10%)	3 (10%)	23 (80%)

HMV = home mechanical ventilation.

Using bivariate correlations, the change in nocturnal pCO_2_ (Δ nocturnal pCO_2_) was positively correlated with the change in SRI scores (Δ SRI; r = .427, p < .05). This suggests that patients who exhibited greater reductions in nocturnal pCO_2_ following treatment also experienced more substantial improvements in their HRQL (Table 5). Baseline demographic and clinical characteristics were not significantly associated with treatment response to HMV (Table 5). No association was found between treatment adherence to HMV and improvements in pCO_2_ or HRQL (Table 5). Comparison of patients with low versus high adherence likewise revealed no significant differences (data not shown). Similarly, no differences were observed between patients who initiated HMV at home and those who initiated it in the hospital (data not shown).

**Table 5.** Exploratory bivariate correlations between baseline characteristics, treatment adherence, and changes in treatment response variables.

	1.	2.	3.	4.	5.	6.	7.	8.	9.	10.	11.	12.
1. Age	-											
2. BMI	.126	-										
3. MRC score on muscle strength	−.528**	.079	-									
4. FVC	−.291	−.168	.644**	-								
5. Daytime pCO_2_	−.060	.013	−.033	−.181	-							
6. Nocturnal pCO_2_	−.065	.188	−.281	−.395*	.342*	-						
7. SRI	−.289	−.384*	−.141	.192	.094	.018	-					
8. Treatment adherence: days used	.088	.004	.183	−.120	−.057	−.140	.011	-				
9. Treatment adherence: use per night	−.028	−.026	.0002	−.057	−.040	.088	.088	.476**	-			
10. Δ SRI	.037	.270	.432*	.268	−.274	−.161	−.199	.183	.055	-		
11. Δ daytime pCO_2_	.084	.096	.250	.235	.593**	−.014	−.127	−.080	−.057	−.022	-	
12. Δ nocturnal pCO_2_	.124	.198	−.101	.003	−119	.323	.003	.143	.036	.427*	.182	-

BMI = body mass index; MRC = Medical Research Council; MIRS = muscular impairment rating scale; FVC = forced vital capacity; pCO_2_ = partial pressure of CO_2_; SRI = Severe Respiratory Insufficiency questionnaire; values represent Pearson correlation coefficients (r) with *p < 0.05, **p < 0.01 (p values are unadjusted for multiple comparisons).

## Discussion

This prospective multicenter study demonstrates that noninvasive HMV is associated with significant improvements in gas exchange and HRQL in patients with DM1 and chronic respiratory failure. Six months of nocturnal HMV significantly improved both daytime and nocturnal gas exchange, as reflected by reductions in pCO_2_ and increase in nocturnal oxygenation. While a positive effect of HMV on gas exchange in DM1 has been previously reported, the evidence primarily came from observational studies in small cohorts.^27–29^ Moreover, in our study, there was a significant increase in HRQL measured with the SRI questionnaire, and improvements in nocturnal gas exchange were associated with better HRQL.

The use of HRQL as outcome measure has not been reported before in this context, although there has been mentioning of beneficial effects of HMV in DM1.^27,30,31^ Unfortunately, we did not observe an effect on symptoms of fatigue and daytime sleepiness, as measured by the FDSS questionnaire. This may be due to the fact that these symptoms are not solely attributable to sleep-disordered breathing and respiratory failure, but likely stem from a more complex and still unclear origin, with existing evidence suggesting a central component.^32,33^ Nevertheless, the majority of the patients (70%) in the current study chose to continue HMV after six months, indicating a favorable overall experience.

Treatment adherence is generally considered an important determinant of the effectiveness of home mechanical ventilation, yet adherence remains challenging in a subset of patients with DM1. In the present study, adherence varied widely, and 10 patients discontinued noninvasive ventilation during the six-month follow-up period. This variability is consistent with previous reports highlighting the burden of long-term ventilatory support in DM1.^6,34–36^ Despite this, significant improvements in gas exchange and HRQL were observed at the group level, and no clear association between adherence metrics and treatment response was identified. These findings should be interpreted cautiously, given the exploratory nature of the analyses and the limitations of adherence data availability across different ventilator platforms. Nonetheless, they suggest that clinically meaningful benefits of HMV may be achieved even in the presence of variable adherence, while underscoring the importance of individualized support strategies to optimize long-term use.

With regard to initiation of HMV, it was previously common practice in the Netherlands to initiate HMV during a hospital admission. However, it has since been demonstrated that home initiation is noninferior to hospital initiation regarding improvement of gas exchange and HRQL.^24^ In our study, both home and hospital initiation were included, yielding comparable outcomes confirming that home initiation is a good alternative for hospital initiation, also in patients with DM1.^37,38^

In DM1, respiratory failure is typically multifactorial, arising from a combination of impaired ventilatory drive, reduced muscle strength, and upper airway obstruction.^5,39,40^ However, the relative contribution of each of these components may vary widely between individuals. Sleep apnea is very prevalent in DM1 which was confirmed in our findings with proven sleep apnea in almost 90% of the patients that underwent a PSG.^41–43^ Because of the additional hypercapnia, these patients were all treated with HMV instead of CPAP, with a positive effect on the AHI. It is plausible that patients with predominant central dysregulation may respond differently to HMV than those in whom respiratory muscle weakness is the primary driver of hypoventilation.^39^ Similarly, the degree of baseline pCO_2_ could influence both physiological and subjective responses to HMV. Unfortunately, due to our relatively small sample size it was not possible to stratify patients based on the severity of respiratory failure, or distinguish between different underlying mechanisms of respiratory failure. Moreover, the small sample size limits the power to detect more subtle effects of predictors of response. The rarity of DM1 limited our ability to include a larger number of patients within the study period, despite a substantially higher number of individuals being screened for eligibility. Therefore, a selection bias cannot be ruled out, particularly since 10 patients (11%) declined to participate. In addition, although medication use was recorded, a subset of patients was using stimulant medication (e.g., modafinil), and analyses were not adjusted for stimulant use. This may have influenced FDSS and other patient-reported outcomes and should be considered a potential confounder. Another limitation of our study is the relatively short follow-up period of six months. Long term results including survival data are not available at this moment. Despite these limitations, this study offers valuable clinical insights into the response to HMV in patients with DM1.

Overall, our findings indicate that the majority of patients responded favorably to HMV. However, the inherent heterogeneity of DM1 remains a significant factor influencing treatment outcomes, including the degree of treatment adherence and the extent of benefit perceived by individual patients. Integrating objective clinical parameters with patient-reported outcomes is essential to determine treatment effectiveness, along with individualized treatment strategies instead of a one-size-fits-all approach. Future research should incorporate larger patient cohorts with a longer follow-up period, and focus on detailed phenotyping at baseline in order to better predict which patients are most likely to benefit from HMV and to refine patient selection criteria.
